# Robustness effect of gap junctions between Golgi cells on cerebellar cortex oscillations

**DOI:** 10.1186/2042-1001-1-7

**Published:** 2011-03-01

**Authors:** Fabio M Simões de Souza, Erik De Schutter

**Affiliations:** 1 Computational Neuroscience Unit, Okinawa Institute of Science and Technology, Okinawa 904-0411, Japan; 2 Theoretical Neurobiology, University of Antwerp, B-2610 Antwerpen, Belgium

## Abstract

**Background:**

Previous one-dimensional network modeling of the cerebellar granular layer has been successfully linked with a range of cerebellar cortex oscillations observed *in vivo*. However, the recent discovery of gap junctions between Golgi cells (GoCs), which may cause oscillations by themselves, has raised the question of how gap-junction coupling affects GoC and granular-layer oscillations. To investigate this question, we developed a novel two-dimensional computational model of the GoC-granule cell (GC) circuit with and without gap junctions between GoCs.

**Results:**

Isolated GoCs coupled by gap junctions had a strong tendency to generate spontaneous oscillations without affecting their mean firing frequencies in response to distributed mossy fiber input. Conversely, when GoCs were synaptically connected in the granular layer, gap junctions increased the power of the oscillations, but the oscillations were primarily driven by the synaptic feedback loop between GoCs and GCs, and the gap junctions did not change oscillation frequency or the mean firing rate of either GoCs or GCs.

**Conclusion:**

Our modeling results suggest that gap junctions between GoCs increase the robustness of cerebellar cortex oscillations that are primarily driven by the feedback loop between GoCs and GCs. The robustness effect of gap junctions on synaptically driven oscillations observed in our model may be a general mechanism, also present in other regions of the brain.

## Background

Oscillations provide a temporal framework for coordination of neural assemblies [1,2], and slow movement, tonic contractions and motor commands are correlated with oscillatory patterns of activity at low frequencies in sensorimotor areas and cerebellum [3,4]. Indeed, local field potential (LFP) oscillations in the 5 to 30 Hz range have been recorded in the hemispheric regions of the cerebellar cortex [5-8], and previous one-dimensional network modeling of the cerebellar granular layer has been successfully linked with a range of *in vivo *oscillation data from the cerebellar cortex [9-12]. *In vivo*, 5 to 30 Hz LFP oscillations are accompanied by phase-locked bursts of multiunit activity representing granule cell (GC) firing, and appear to be generated at the level of the granular layer [6].

**Figure 1:**
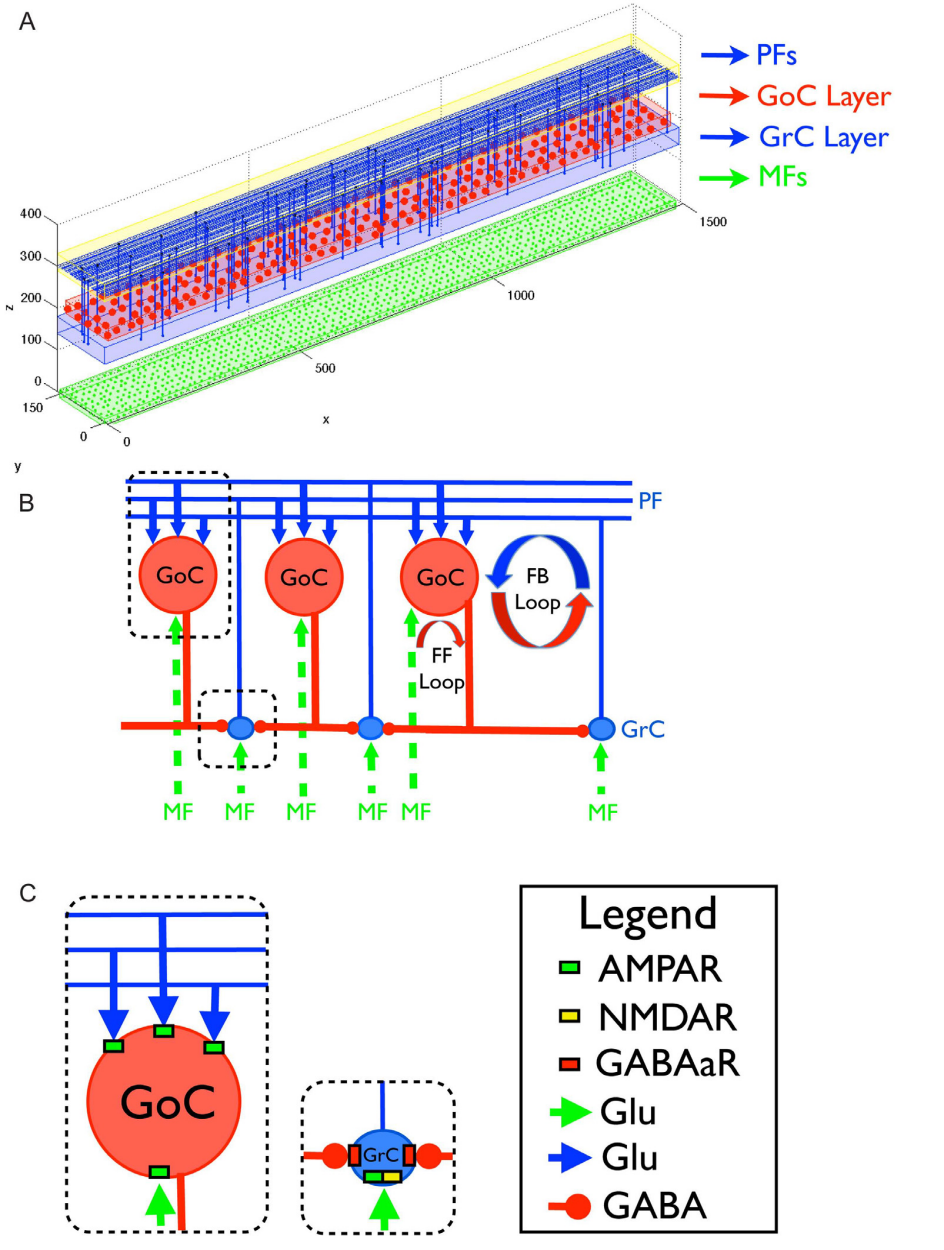
**Neural network topology**. Structure of the network model. **(A) **The actual spatial location of neurons in their corresponding two-dimensional layers is shown. Mossy fibers (MFs) are shown in green, Golgi cells (GoCs) in red and granule cells (GCs) and parallel fibers (PFs) in blue. Only 1% of the GCs and PFs are displayed for better visualization. **(B) **Schematic diagram illustrating the connectivity between the layers. MFs (green) excite both GCs (blue) and GoCs (red). The axons of GCs form ascending fibers, which bifurcate in the PF fiber layer and spread in each direction of the *x *axis. These PFs excite the GoCs along the way. By contrast, GoCs inhibit the GCs in their vicinities. Two inhibitory circuits driven by the excitatory MF inputs emerge from this synaptic organization. One is a feedforward (FF) inhibitory circuit and the other is a feedback (FB) inhibitory circuit. The FF circuit works through the MF-GoC-GC pathway, whereas the FB circuit works through the MF-GC-PF-GoC-GC pathway. **(C) **Inset showing the neurotransmitters and synaptic receptors used by each modeled synaptic connection. The GoC model has α-amino-3-hydroxy-5-methyl-4-isoxazolepropionic acid receptors (AMPAR), which are activated by MF or PF glutamatergic (Glu) terminals. The GC model has AMPAR and N-methyl-D-aspartic acid receptors (NMDAR; activated by MF Glu terminals), and GABAa receptors (GABAaR) (activated by GABAergic terminals (GABA) coming from the nearby GoCs).

The basic cerebellar cortex circuitry responsible for the generation of oscillations is driven by mossy fibers (MF) that excite both GCs [13,14] and Golgi cells (GoCs) [15-17]. The axons of the GCs form ascending fibers that bifurcate in both directions in the parallel fiber (PF) layer [18,19]. These PFs excite GoCs along their way. By contrast, GoCs are the only source of inhibition for the GCs in their vicinities [20]. Two inhibitory loops driven by the excitatory MF inputs emerge from this synaptic organization [21] (Figure 1B): a feedforward (FF) inhibitory loop and a feedback (FB) one. The FF loop works through the MF-GoC-GC pathway. MFs excite GoCs that then inhibit GCs. The FB loop works through the MF-GC-PF-GoC-GC pathway. MFs excite GCs that then excite GoCs that will inhibit GCs.

Because Golgi interneurons do not inhibit each other, they were considered as independent units until the discovery that they express connexins and pannexins [22-27] and are electrically coupled by gap junctions [28,29].

Many functions have been attributed to gap junctions in neural networks in general [30-32]. In particular, they are known for generating synchrony in networks of inhibitory neurons [33-37], but it has also been suggested that they could desynchronize under particular conditions, such as in the presence of sparse MF inputs [29]. The recent discovery that gap junctions between GoCs receiving excitatory drive by intrinsic or tonic depolarization may cause oscillations by themselves [28] raises the question of how gap-junction coupling affects synaptically driven GoC and granular-layer oscillations, an issue that has not yet been addressed in modeling or experimental studies.

To investigate this question, we developed a novel two-dimensional computational model of the GoC-GC circuit, with and without gap junctions between GoCs. We systematically explored the behavior of these networks for different input and synaptic feedback loop strengths.

## Methods

The model was constructed and numerically solved with the NEURON simulator (version 7.1) [38]. The NEURON code used to generate the network is available at the ModelDB database http://senselab.med.yale.edu/ModelDb/.

### Model GoCs

**Figure 2:**
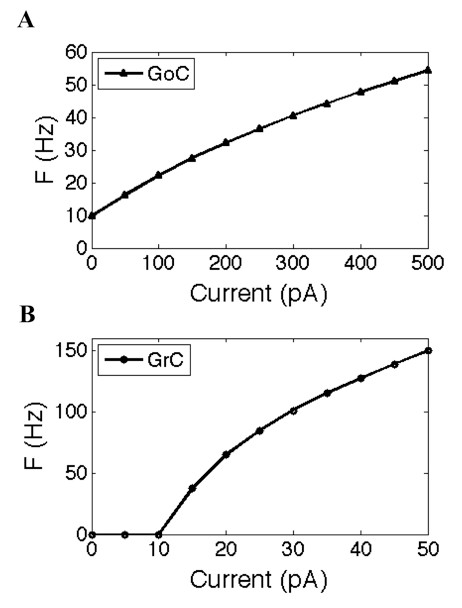
**Single-cell firing frequency responses to input current**. **(A) **Golgi cell (GoC) frequency versus current (F/I) curve; **(B) **granule cell (GC) F/I curve. Mean firing frequency was measured over a 10 second simulation.

The GoC model is identical to a previously published model [39], but without the compartmental axon. It consists of four compartments including a soma and three dendrites, with an input resistance measured at the soma of 159 MΩ. It has 12 voltage-dependent ionic channels reproducing GoC intrinsic firing and responsiveness to somatic current injection [40]. We adopted a resting membrane potential of -60 mV and a passive leakage current with reversal potential at -44.5 mV. The frequency versus current (F/I) curve of a single GoC model is shown in Figure 2A. Note that GoCs have spontaneous (pacemaking) firing at 0 pA, and increasing firing rates upon strong current injections.

### Model GCs

The GC model is based on previously published models [41-44]. Briefly, we reduced a multicompartmental model [44] to a single compartment model that is suitable for use in the neural network model. The GC model is a spherical compartment with a diameter of 11.8 μm and input resistance of 1.62 GΩ. It is somewhat similar to a previous single-compartment model [41], but using upgraded channel densities and ionic channels [42,43]. The F/I curve response of the GC model is shown in Figure 2B. Note that GCs have no spontaneous firing, and their firing threshold is around 10 pA. These electrotonic compact neurons can fire at 150 Hz in response to a current injection of 50 pA.

The model neurons were validated to reproduce *in vitro *preparations at room temperature [40,41]. Because we verified that the neuron models work best in that temperature range [41-43] and do not have robust behavior at higher temperatures, we used a temperature of 23°C for all model neurons.

### Network description

The model has a spatial dimension of 0.15 × 1.5 mm, and it is composed of three two-dimensional (2D) matrices, respectively representing the MF, GC and GoC layers (Figure 1). We choose a 2D structure as an intermediate level between the one-dimensional (1D) and three-dimensional (3D) approaches, allowing us to run computationally less expensive simulations while preserving the basic characteristics of the biological networks. The 2D structure is more accurate than the previously used 1D approach [9], allowing us to connect GoCs along multiple axes by gap junctions. It also has a smaller number of cells than in the 3D approach [45,46], allowing us to simulate larger pieces of cerebellar tissue (0.15 × 1.5 mm). In this way, the synaptic weights, delays, and gap-junction conductances (Gj) can still be associated with realistic Euclidian distances between the neurons.

The network model has a total of 9,225 cellular units and 935,063 synaptic connections distributed along the layers. The three layers are composed of 900 (10 × 90) spike-generator MFs, 8100 (30 × 270) conductance-based GCs and 225 (5 × 45) conductance-based GoCs. There are 32,547 MF synapses on GC α-amino-3-hydroxy-5-methyl-4-isoxazolepropionic acid receptors (AMPAR) and N-methyl-D-aspartic acid receptors (NMDAR), 20,822 MF synapses to GoC AMPAR, 783,017 GC synapses to GoC AMPAR, and 66,130 GoC synapses to GC γ-aminobutyric acide (GABA)_a _receptors. These numbers arise from several parameters based on published data available [13,14,17,47,48] or from previous computational models [9], as described in more detail below.

Some parameters were adopted because of computational limitations. For example, there are about 400 GCs for every GoC in the rat cerebellum [49,50], but for modeling long pieces of the cerebellar cortex (1.5 mm) it is not practical to simulate 90,000 GCs. Therefore, we used a more reasonable ratio of 36 GCs for every GoC, which was sufficient for obtaining synaptically driven oscillations.

To avoid artificial synchrony in the network, several model parameters were randomized. This included the leak current, membrane area and initial membrane potential of each neuron, which were varied by ± 20% around their mean value. The X and Y spatial coordinates of the neurons were also randomized by ± 20% to obtain more physiological spatial distributions and cause an effective randomization of distance-dependent synaptic and gap-junction strengths (see Additional file 1, Figure S1 and Figure S2).

### Synaptic conductances and spike conduction times

Synaptic conductances were modeled by double exponential functions according to Maex *et al*. [9]. The AMPAR conductance model has a rise time constant τ_1_= 0.03 ms, decay time constant τ_2 _= 0.5 ms and reversal potential E_rev_= 0 mV [51,52]; the NMDAR conductance model has τ_1 _= 1 ms, τ_2 _= 13.3 ms, E_rev _= 0 mV [53]; and the GABAaR conductance model has τ_1 _= 0.31 ms, τ_2 _= 8.8 ms and E_rev _= -75 mV [54]. The activation of at least two MFs was required to elicit a GC spike [53].

The spike-conduction time defines the delay that a presynaptic spike takes to propagate from the axon hillock to the axon terminal of the presynaptic neuron and then activate the postsynaptic neuron, and it depends on the speed of the propagation and the distance between the presynaptic and postsynaptic neurons.

The propagation delays and the synaptic conductances were scaled according the distance between the presynaptic and postsynaptic neuron. Delays increased linearly with the Euclidian distance of the fibers, assuming a uniform speed of action potential propagation of 0.5 m/s [55,56]. The mean ± SD delay was 0.026 ± 0.009 ms for MF to GC AMPAR and NMDAR, 0.312 ± 0.148 ms for MF to GoC AMPAR, 1.308 ± 0.695 ms for GC to GoC AMPAR, and 0.0758 ± 0.028 ms for GoC to GC GABA_a_R (see Additional file 1, Figure S3).

The macroscopic conductance calculated from inhibitory/excitatory postsynaptic currents (IPSCs/EPSCs) recorded from voltage-clamp experiments to monosynaptic stimulation is around 124 picoSiemens (pS) for MF to GC [14], 833 pS for MF to GoC [17], and between 137 pS [14] and 420 pS [17] for GoC to GC. The estimated PF to GoC macroscopic conductance is around 666 pS [17]. Except for the PF to GoC connections, which were constant along PFs [52], all other synaptic conductances decreased exponentially with the Euclidian distance (decay parameter) being equal to 0.01/μm, which implied a glomeruli-like pattern of connectivity [57]. The scaled mean ± SD synaptic conductances for MF to GC AMPAR and NMDAR were 2.28 ± 0.11 nS and 0.198 ± 0.009 nS, MF to GoC AMPAR 0.706 ± 0.506 nS, GoC to GC GABA_a_R 0.968 ± 0.140 nS, and PF to GoC AMPAR 2.588 nS (see Additional file 1, Figure S4).

### Network connectivity

The connectivity of the network is based on the convergence and divergence patterns of the cerebellar cortex neurons (see Additional file 1, Figure S5) [48,58-60]. We adopted a mean convergence of four MF on each GC [13,14]. For MF to GoC, the numbers are not well known, but are >4 [17]. Considering the large GoC dendritic tree [20,52], we selected an average of 100. One MF can supply excitatory synapses to about 400 GCs [60], but because we used smaller GCs:GoC ratios, then each MF diverged to 39 GCs and 25 GoCs. The mean convergence of PF to GoC was 4000 [58,60], and about eight GoC connections were made on each GC [13]. GoCs extend broadly branching axons to up to around 5700 GCs in cats [49], and single GoCs presumably can trigger inhibition in thousands of GCs [17,47], but, once again, because of our reduced GCs:GoC ratios, every modeled GoC diverged to about 350 GCs, and each PF diverged to 100 GoCs.

### Gap junctions

**Figure 3:**
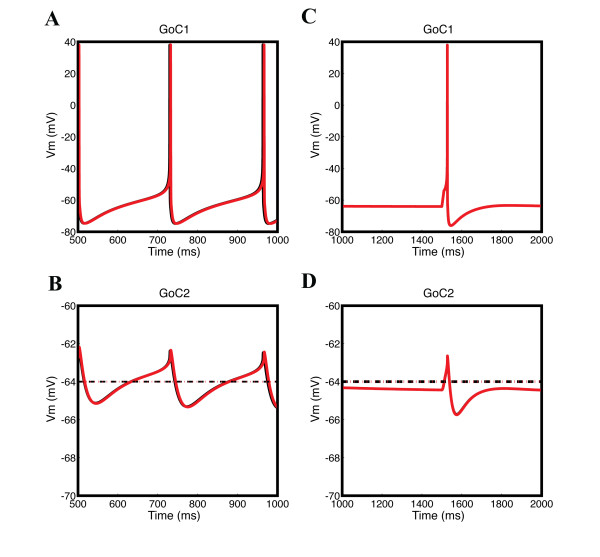
**Electrical coupling between two Golgi cells (GoCs) connected by gap junctions either on the soma (red) or on the dendrite (black)**. Membrane potentials recorded from the soma of two gap junction-coupled GoCs in response to either **(A, B) **mossy fiber (MF) synaptic input at 10 Hz or **(C, D) **to a depolarizing current pulse. GoC1 and GoC2 were coupled by gap junctions either on the soma (red) or in the dendrite (black). **(A, C) **On the top, GoC1 is spiking and transmits to **(B, D)** thenon-spiking GoC2 below. In (C), pacemaker firing in GoC1 was suppressed with a hyperpolarizing current of -0.1 nA and a spike was evoked by a 100 ms pulse of 0.5 nA at 1500 ms. The spikes of GoC2 were always suppressed by setting all the sodium channel conductances to zero. Note **(B, D) **that the fast components of the spike (rising phase, peak and falling phase) are filtered more than the slower ones (undershoot), and that gap junction location does not matter much (red and black curves are superimposed). Dotted lines indicate the GoC resting membrane potential.

The membrane potential response of a non-spiking GoC coupled by gap junctions to a spiking GoC is composed of a depolarizing component related to the rising and falling phases of the action potential, and a hyperpolarizing component linked to the undershoot phase [28]. This coupling effect of gap junctions is illustrated by two coupled GoC models showing one spiking GoC stimulated either by MF input (Figure 3A) or by a current pulse (Figure 3C), and transmitting the depolarizing and hyperpolarizing components of spikes to another GoC (Figure 3B, D), replicating experimental observations. Note that gap junctions placed between dendrites (Figure 3, black lines) or between somata (Figure 3, red lines) produced the same effect in our GoC models.

The gap junctions show low-pass filtering effects [28]: fast components of the spike (rising phase, peak and falling phase) are more filtered than the slower ones (undershoot) by the non-spiking GoC coupled by gap junctions (Figure 3B, D).

Moreover, in accordance with experimental data [28], we modeled the Gj decaying with distance between the soma of GoCs in the network (decay parameter 0.03/μm). In addition, we randomized the Gj by 60% to reproduce the physiological variability [28]. For short distances between GoCs, the (Gj) ranged from 0.25 to 1.25 nS [28] (see Additional file 1, Figure S1).

### Stimulation pattern

The network was stimulated with spatially uniform random MF spikes. The MF input firing rates followed a normal distribution with center at a given stimulation frequency. In this way, the network was stimulated with MF mean firing rates ranging from 1 to 100 Hz. For each simulation, the model ran without MF inputs for 1 second, and then MF inputs were presented for another 1 second. The specific mean ± SD of the Gaussian distributions for the MF firing rates were 1 ± 0.71, 5 ± 1.90, 10 ± 3.00, 15 ± 3.33, 20 ± 4.28, 40 ± 6.16, 70 ± 7.57 and 100 ± 8.72 Hz.

In some cases, our simulations achieved unnaturally high sustained GoC firing frequencies of up to 120 Hz during strong MF inputs, which we attribute to the absence of modulation in our simulated MF input. GoCs fire transiently at high rates after stimulation *in vivo *[16]. The high sustained GoC firing rates during strong MF inputs are not a limitation of the model itself, but rather of the steady-state input condition that we required to analyze oscillations under spatial and temporal steady-state conditions.

In some simulations, we analyzed the resonance frequency of the GoC layer in response to injection of sinusoidal current waves in the soma of each GoC. In this case, both GoCs and GCs received background Gaussian MF inputs at 7 ± 2.86 Hz to introduce noise into the network. The peak of the sinusoidal currents was set at ± 0.1 nA and the frequency was set at 1 Hz, 5, 10, 15, 20, 30, 40, 50, 60, 70, 80, 90 and 100 Hz. In addition, the resonance frequency of a larger version of the network elongated to 6 mm was tested in response to the following Gaussian distributions of MF firing rates: 1 ± 0.71, 5 ± 1.90, 10 ± 3.00, 15 ± 3.33, 20 ± 4.28, 30 ± 4.92, 40 ± 6.15, 45 ± 6.34, 50 ± 6.63, 55 ± 6.86, 60 ± 7.17, 65 ± 7.39, 70 ± 7.57, 80 ± 8.10, 90 ± 8.24 and 100 ± 8.72 Hz.

### Data analysis

Border effects of the network were avoided by using only the neurons in the center of the network for the analysis. The borders were removed until the spatial distributions of cellular firing frequencies were uniform along the *x *and *y *axes of the network. In this way, the specific matrix dimensions considered for the analysis were 6 × 82 (492) for the MF layer, 18 × 246 (4428) for the granular layer and 3 × 41 (123) for the GoC layer.

We then recorded the spike timings of all neurons in the network for each simulation, and used the cellular indexes and the spike timings to construct the raster plots. We averaged the number of spikes of each firing neuron across the stimulation time to obtain the mean firing rates (MFRs) for each network layer. We used 1 millisecond bins to calculate the total number of spikes of the each layer of network per bin to produce the population spike timing histograms (PSTHs), then calculated the spectral frequencies of the PSTH oscillations for the GoCs using Morlet wavelet analysis [61] (see Additional file 1, Figure S6).

For the analysis of the amplitudes of the oscillation cycles and the latencies for the occurrence of the oscillations, the PSTHs for the GoCs were filtered by a low-pass Butterworth filter of order 5 with cut-off frequency of 500 Hz to remove the background noise from the signals. All the network responses were analyzed for 1 second of simulation in the presence of the stimulus (1000 to 2000 ms).

## Results

All network conditions were compared for the presence or absence of gap junctions. Raster plots and PSTHs were obtained for each condition, and the oscillation frequency of the PSTHs was analyzed.

**Figure 4:**
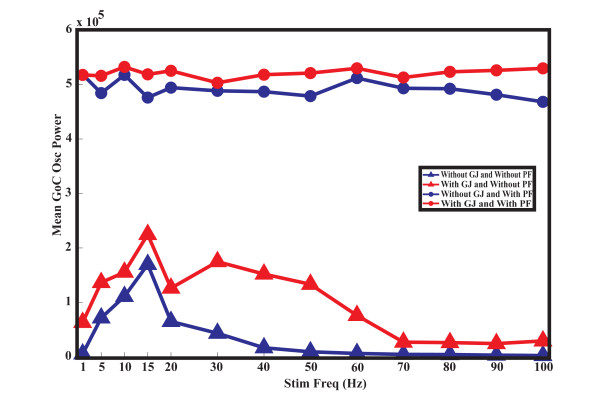
**Mean Golgi cell (GoC) oscillation power versus sinusoidal current input frequency**. The mean power of the GoC population spike timing histograms (PSTHs) for the network without gap junctions is in blue, and with gap junctions is in red. The response of the network without parallel fibers (PFs) (feedforward configuration) is shown with triangles, and the response with PFs (feedback configuration) is shown with circles.

The granular layer works as a resonator that is enhanced to oscillate around a characteristic frequency [62]. Therefore, we analyzed (similar to the approach described previously [28]), the resonance frequency of the GoC layer in response to injection of sinusoidal waves into the soma of each GoC. Both GoCs and GCs received low-frequency random MF input to introduce noise into the network. In the absence of PF inputs, the GoC network without gap junctions had a resonance frequency of 15 Hz (Figure 4, blue triangles). The presence of gap junctions did not change this resonance frequency, but it increased the power of the oscillations (Figure 4, red triangles). The presence of gap junctions also induced a secondary gamma band peak around 30 Hz. These results are consistent with previous modeling studies [28]. When PFs were introduced into the network, resonance was lost and the power of oscillations increased in response to all frequencies of sinusoidal current, both in the presence (Figure 4, red dots) and absence of gap junctions (Figure 4, blue dots).

**Figure 5:**
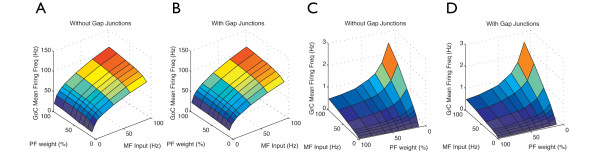
**Mean firing rate of the neurons in the network**. **(A, B) **Golgi cell (GoC) layer mean firing rate; **(C, D) **GC layer mean firing rate. **(A, C) **network without gap junctions between GoCs; **(B, D) **network with gap junctions between GoCs. The *x *axis = MF input rate (Hz); *y *axis = parallel fiber (PF) synaptic weight (%); and *z *axis = mean firing frequency (Hz).

After studying the responses of the network to sinusoidal waves, the network was stimulated directly by synaptic inputs with varying rates of MF input on GoCs and GCs. We also investigated the influence of the strength of the PFs on the network activity in the presence and absence of gap junctions between GoCs. Initially, we tested the influence of the synaptic inputs on the MFRs of GoCs and GCs. MFRs increased with increasing MF rates and PF strengths (Figure 5A, C), and gap junctions did not affect the MFRs of the neurons in the network (Figure 5B, D; see Additional file 1, Figure S7). GC MFRs were 0.5 to 3 Hz and GoC firing rates were 20 to 120 Hz (see Methods). *In vivo*, GC and GoC MFRs are 2 to 7 Hz [6,63-65].

**Figure 6:**
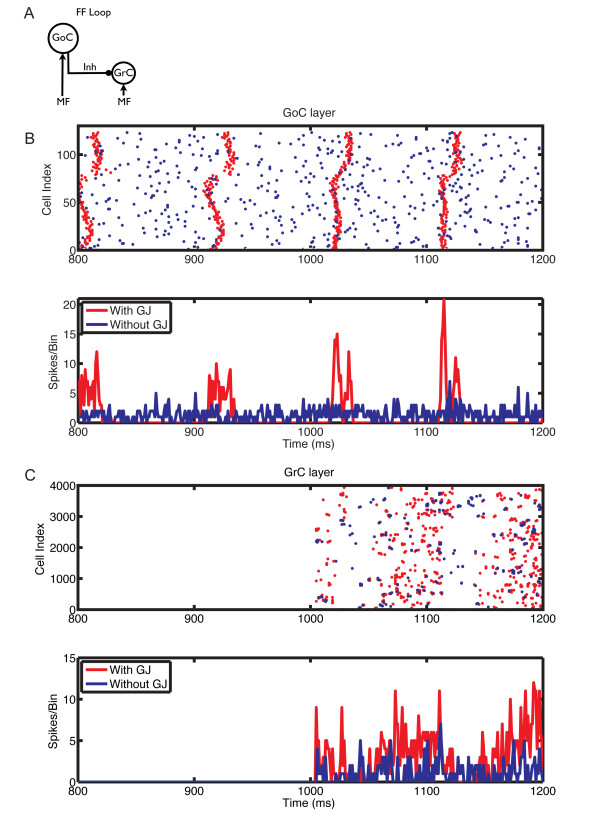
**The influence of gap junction between Golgi cells (GoCs) on the responses of the network in the feedfoward loop configuration, with mossy fiber (MF) input at 5 Hz**. **(A) **Feedforward loop configuration: network without parallel fiber (PF) input to GoCs. **(B) **Raster plot (top panel) and population spike timing histogram (PSTH) (bottom panel) of the GoC layer with (red) and without (blue) gap junctions. Each dot in the raster plot is a spike. MF mean firing rates (MFRs) inputs at 5 Hz were turned on at the instant of 1000 ms. **(C) **Same as (B) but for the GC layer.

**Figure 7:**
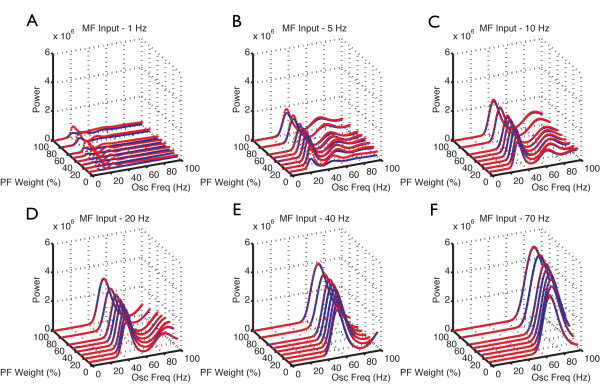
**Power spectral frequency of the Golgi cells (GoCs) population spike timing histograms (PSTHs) in the presence of varying mossy fiber (MF) input rates and parallel fiber (PF) synaptic weights**. Network responses of the GoC layer with (red) and without gap junctions (blue) between GoCs. Power spectral density for each PF strength and MF input rate (A to F) for the regular network model of length 1.5 mm. X axis: Oscillation frequency (Hz). The *y *axis = PF synaptic weight (%), *z *axis = normalized power. MF rate at **(A) **1 Hz, **(B) **5 Hz, **(C) **10 Hz, **(D) **20 Hz, **(E) **40 Hz, (**F) **70 Hz.

**Figure 8:**
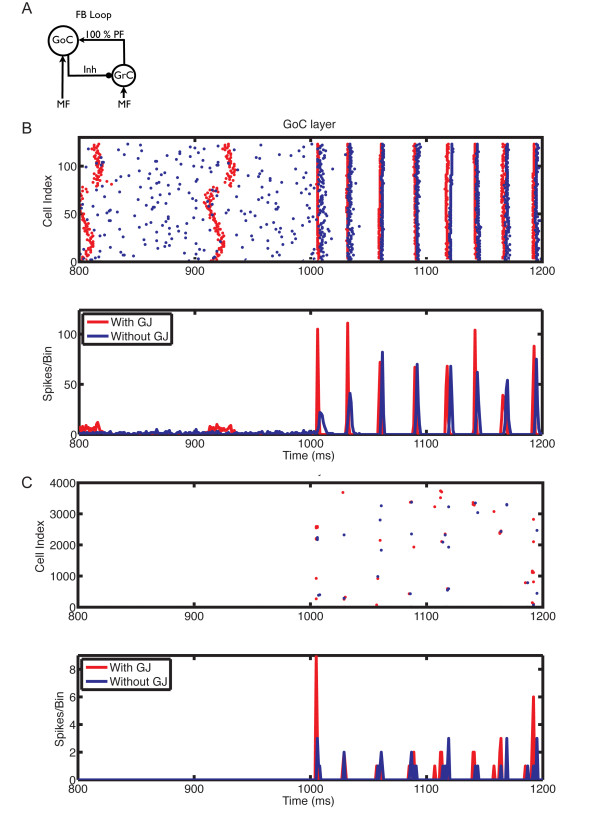
**The influence of gap junctions between Golgi cells (GoCs) on the responses of the network in the feedback loop configuration, with mossy fiber (MF) input at 5 Hz**. **(A) **Feedback loop configuration: network with strong PF inputs and 100% synaptic weight. **(B) **Raster plot (top panel) and population spike timing histogram (PSTH) (bottom panel) of the GoC layer with (red) and without (blue) gap junctions. Each dot in the raster plot is a spike. MF mean firing rate (MFR) inputs at 5 Hz were turned on at the instant of 1000 ms. **(C) **Same as (B) but for the GC layer.

**Figure 9:**
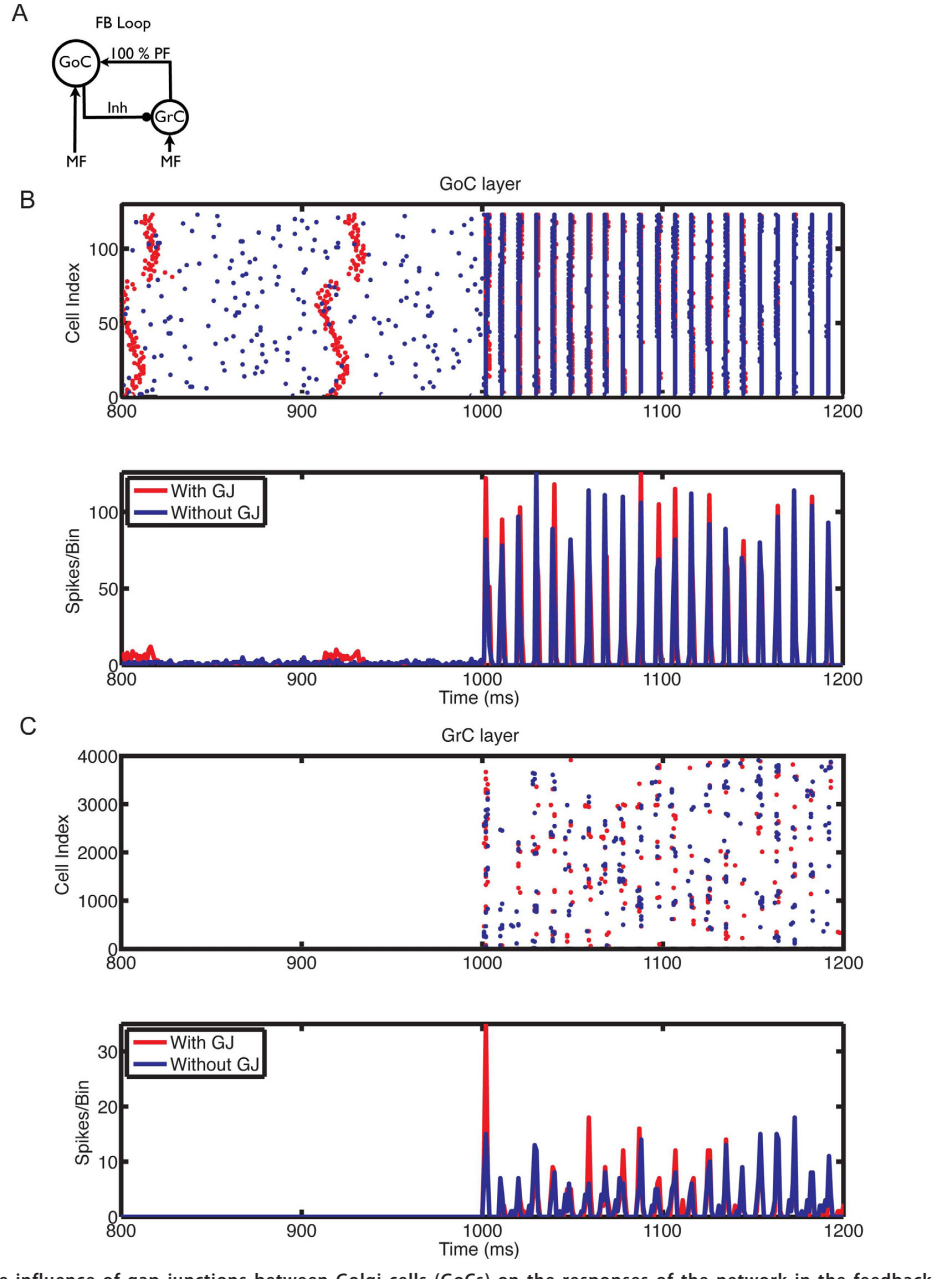
**The influence of gap junctions between Golgi cells (GoCs) on the responses of the network in the feedback loop configuration, with mossy fiber (MF) input at 100 Hz**. **(A) **Feedback loop configuration: network with strong PF inputs and 100% synaptic weight. **(B) **Raster plot (top panel) and population spike timing histogram (PSTH) (bottom panel) of the GoC layer with (red) and without (blue) gap junctions. Each dot in the raster plot is a spike. MF mean firing rate (MFR) inputs at 100 Hz were turned on at the instant of 1000 ms. **(C) **Same as (B) but for the GC layer.

**Figure 10:**
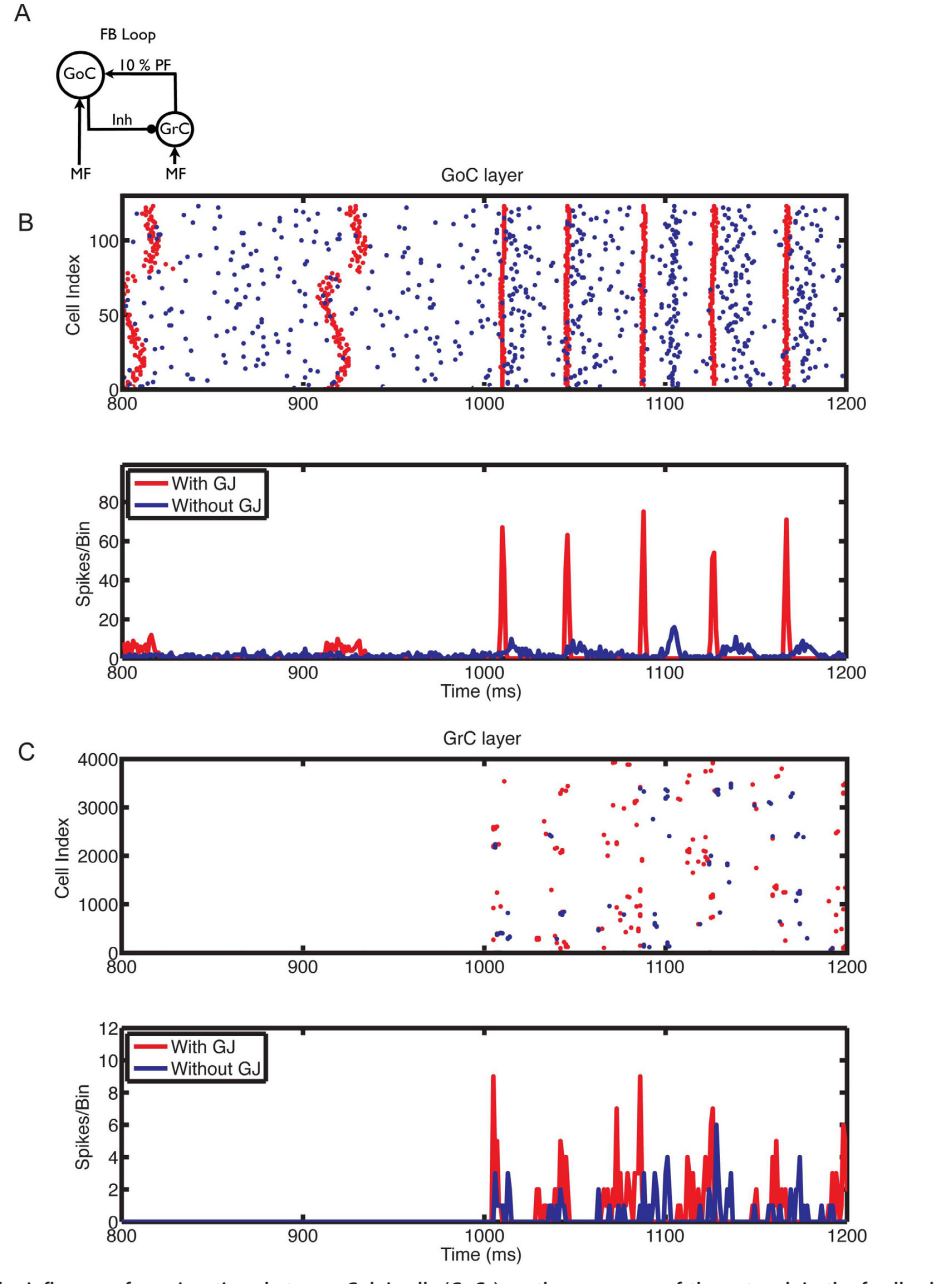
**The influence of gap junctions between Golgi cells (GoCs) on the responses of the network in the feedback loop configuration with weak PF inputs and mossy fiber (MF) input at 5 Hz**. **(A) **Feedback loop configuration: network with weak PF inputs and synaptic weight reduced to 10%. **(B) **Raster plot (top panel) and population spike timing histogram (PSTH) (bottom panel) of the GoC layer with (red) and without (blue) gap junctions. Each dot in the raster plot is a spike. MF mean firing rate (MFR) inputs at 5 Hz were turned on at the instant of 1000 ms. **(C) **Same as (B) but for the GC layer.

We next investigated the effect of gap junctions on network oscillations under different conditions of MF activation and PF-connection strengths. We previously showed that the granular layer oscillates when the PF input to GoCs is strong (feedback configuration) and it is activated by MF input; conversely, in the absence of PF input (feedforward configuration) or of MF activation, GoC firing is desynchronized [9]. The latter is no longer true in the presence of gap junctions between GoCs (Figure 6). In the feedforward configuration, gap junction-coupled GoCs had a strong tendency to generate spontaneous slow and poorly synchronized GoC oscillations (Figure 6B, 800 to 1000 ms). Activation of MF input improved synchronization, and slightly increased oscillation frequency. Whereas gap junctions have a pronounced effect in the feedforward configuration, their effect is more subtle in the feedback configuration of the network, which more closely approximates the *in vivo *behavior of the granular layer [66-68] (Figure 7, Figure 8, Figure 9, Figure 10).

In the presence of MF and PF input, the frequency and the power of the network oscillations increased with the strength of the PFs and with the rate of MF inputs (Figure 7). The power spectral density of the oscillations showed a primary band at 15 to 30 Hz, and a secondary gamma band at 30 to 100 Hz for MF inputs at 1, 5 and 10 Hz (Figure 7). The frequency and the power of the secondary band also increased with the PF strength and with the MF MFRs, and gamma oscillations became the primary band for higher MF MFRs (>10 Hz).

No resonance was observed in the regular network of 1.5 mm length. This can be explained by the long delays in the propagation of the spikes along the PFs, required for the occurrence of resonance in cerebellar cortex models [11,32]. Because the PFs did not reach their maximum lengths (2.5 mm in each direction [19]) in the regular network, this implied resonance frequencies at non-physiologically high frequencies that were beyond the range we explored. To overcome this limitation, we ran control simulations with a network that had a length of 6 mm, providing enough PF length for the emergence of resonance that occurred at the expected frequency of 65 Hz [11,32] with 100% of PF synaptic weight (see Additional file 1, Figure S8). The presence of gap junctions did not change the primary resonance frequency of the elongated network.

The oscillations were primarily driven by the synaptic feedback loop between GoCs and GCs, and the presence of gap junctions did not change their frequency, but increased their power, particularly with weak PF synaptic weights and slower MF inputs (Figure 7A-C). This is exemplified by the raster plots (Figure 7, Figure 8, Figure 9, Figure 10). Before the activation of the MFs, there was no spontaneous GC firing (Figure 8C, 800 to 1000 ms), and therefore there was no PF input onto the GoCs. During this period, the only activity in the network came from the pacemaker spiking activity of GoCs (Figure 8B), which have a tendency to synchronize in the presence of gap junctions (red dots and traces). By contrast, when MFs were activated, GCs started firing, therefore GoCs received both MF and PF inputs (Figure 8B, 1000 to 1200 ms), and in turn inhibited the GCs. The result of the activation of this feedback loop was the emergence of synaptically driven synchronous oscillations in the neural network (Figure 8, Figure 9, Figure 10, 1000 to 1200 ms). In the case of 100% PF synaptic weight, the first oscillatory cycles were stronger in the presence than in the absence of gap junctions (Figure 8 and Figure 9). This implies that during these initial cycles, the neurons are more synchronized in the presence than in the absence of gap junctions; afterwards, their behavior equalizes, and the PSTH amplitudes of the cases with and without gap junctions become similar. However, the oscillations in the presence of gap junctions had a permanent change in phase, resulting from the fact that the latency for the occurrence of the first oscillatory cycle was smaller in the presence of gap junctions, whereas the frequency of the oscillations was the same in both cases (Figure 8). The robustness effect observed in the initial oscillatory cycles in the case with 100% PF synaptic weight (Figure 8) was more pronounced in the presence of weak (10%) PF synaptic weights (Figure 10). In this case, the weak synaptic feedback was sufficient to maintain the oscillations in the absence of gap junctions, but the presence of gap junctions strongly increased the synchrony of firing, resulting in a constant higher power for the oscillations (Figure 7B). Again, the first oscillatory cycle occurred earlier in the presence of gap junctions, but the frequency of the oscillations was still the same in both cases, implying a change of their phase (Figure 10).

The other synaptic weight of the feedback loop, that of the inhibitory GoC-GC GABA_a _receptors, had much less effect. This parameter is important to maintain the occurrence of oscillations, but it had little effect on the robustness effect. The minimum synaptic weight required to avoid disruption of the oscillations in the cerebellar cortex model increased along with the MF MFRs, and it was independent of the presence or absence of gap junctions between GoCs (see Additional file 1, Figure S9).

**Figure 11:**
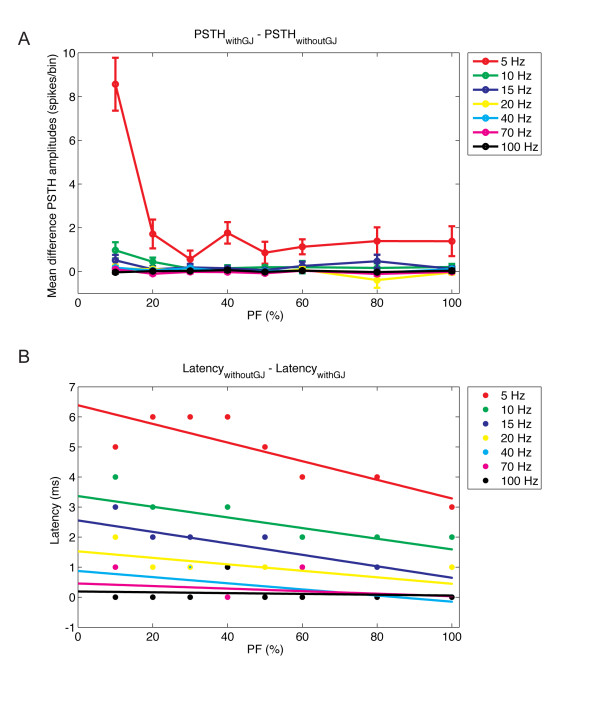
**Quantification of the robustness effect of gap junctions**. **(A) **Mean difference in population spike timing histogram (PSTH) amplitudes (spikes/bin) in the Golgi cell (GoC) layer with or without gap junctions for different strengths of parallel fiber (PF) connections and mossy fiber (MF) input rates. The mean and standard deviation were obtained from the amplitude of the oscillation cycles occurring within the interval of 1000 to 2000 ms. **(B) **Difference between the latency (ms) for the occurrence of the first oscillatory cycle after MFs were turned on in the network without or with gap junctions.

We conclude that in the physiologically more realistic feedback condition, gap junctions mainly increase the robustness of the oscillations caused by the synaptic feedback. This robustness effect was quantified (Figure 11A) as the mean difference of the PSTH amplitude between networks with and without gap junctions. The stronger difference was observed in the case of low MF input (MFRs at 5 Hz), and the mean difference between the PSTH amplitudes decreased as the MF MFR increased. In addition, the mean difference in the PSTH amplitudes decreased with increasing PF synaptic weights (most pronounced at <30%). However, even in the cases where the robustness effect was limited to the initial oscillatory cycles (Figure 8 and Figure 9), it was sufficient to affect the latency for the occurrence of the first oscillatory cycle (Figure 8, Figure 9, Figure 10 and Figure 11B), and therefore permanently affect the phase of the oscillations. The difference between the latency for the occurrence of the first oscillatory cycle in the network without or with gap junctions (Figure 11B) showed a similar inverse relationship with the MF MFR and the PF synaptic weights to that of the difference in PSTH amplitude (Figure 11A). The presence of gap junctions between the GoCs reduced the latency for the occurrence of the first oscillatory cycle by several milliseconds over a wide range of input conditions.

**Figure 12:**
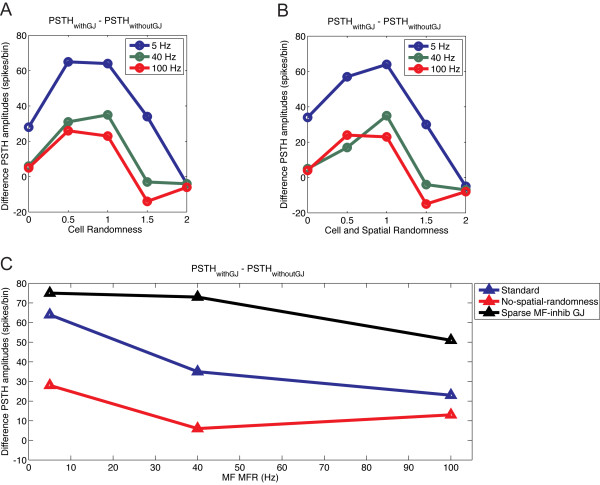
**Effect of randomization on the robustness effect of gap junctions**. Robustness effect was quantified as in Figure 11A. **(A) **Effect of changing the randomness in the leak currents and cellular diameters (cell randomness) relative to standard (feedback configuration with 20% parallel fiber (PF) synaptic weight). **(B) **Effect of combined changes to the cell-randomness and the randomness in the spatial position of the cells relative to the standard. **(C) **Effect of the lack of spatial randomness (red) or of sparser MF inputs onto Golgi cells (GoCs)(black) compared with standard (blue).

The robustness effect of gap junctions compensates for the randomization of elements in the network model, which was introduced to mimic biological variability. We next examined whether the robustness effect was affected by the levels of randomization (Figure 12; see Additional file 1, Figure S2). A representative situation with 20% PF synaptic weight, producing an intermediate robustness effect, was used as the standard case for comparison. We diminished or increased the cellular randomness (that is, the leak current and diameter of soma; Figure 12A) or the spatial and cellular randomness together (Figure 12B). Intermediate spatial and cellular randomness favored the occurrence of the robustness effect, but too much or too little randomness reduced it. Too much randomness disorganized the GoC spontaneous oscillations in the presence of gap junctions, and reduced the robustness effect in the presence of MF inputs; too little randomness increased the spontaneous synchrony of GoCs in the absence of gap junctions. Because the synchrony levels were already naturally very high, gap junctions did not make much difference, reducing the robustness effect. Abolishing spontaneous spiking of GoCs [28] similarly increased synchrony and decreased the robustness effect (results not shown). There was thus a tuning curve for cellular and spatial randomness for the occurrence of the robustness effect (Figure 12A and 12B). In addition, the robustness effect of gap junctions was diminished when the spatial randomness alone was suppressed (Figure 12C).

Finally, we tested whether a sparser MF synaptic input to GoCs, which was used in another recent modeling study [29] and which is able to desynchronize GoCs coupled by gap junctions in the absence of PFs (see Additional file 1, Figure S10), would abolish the robustness effect observed in the presence of PFs. Unexpectedly, the robustness effect increased in the presence of sparse MF and regular PF inputs (Figure 12C; see Additional file 1, Figure S11 and Figure S12). Thus, the robustness caused by gap junctions is likely to occur in a large number of biologically relevant situations.

## Discussion

Many functions have been attributed to gap junctions in neural networks in general [31,32], but their function in the cerebellum is still unclear. Recently, Dugué *et al*. [28] proposed that gap-junction coupling mediates tunable low-frequency oscillations and resonance in the cerebellar GoC network, but they tested their hypothesis in an isolated GoC layer model that uses very simplified neuron models. Our modeling results of the complete network, using state-of-art conductance-based models [39,44], suggest a novel function for gap junctions between GoCs, in improving the robustness of cerebellar cortex oscillations that are primarily synaptically driven.

Our results in the network without PF connections confirmed the previous findings of Dugué *et al*. [28] (Figure 6). In contrast, our modeling results in a more realistic network with PF connections suggest that gap junctions between GoCs increase the amplitude of the cerebellar cortex oscillations without affecting their frequency, which is primarily driven by the synaptic feedback loop between GoCs and GCs as described previously [9,11]. This robustness effect of gap junctions changes the timing of the first oscillatory cycles and improves their synchronization (Figure 8, Figure 9, Figure 10), an effect that is more pronounced at low MF input rates and weak PF synaptic weights.

In our model, the robustness effect of gap junction vanished when the membrane potential of the neurons was homogeneous. This happened when the intrinsic cellular properties and spatial variability of the network were abolished and GoCs started firing artificially at very high synchrony, whether gap junctions were present or not. However, the robustness effect was also suppressed when the network randomness was too high, which resulted in reduction in GoC spontaneous synchronization in the presence of gap junctions. These results (Figure 12A, B) suggest that the robustness effect of GoC gap junctions on the granular layer network is optimized to work at biologically relevant intermediate levels of cellular and spatial randomness.

Oscillations observed in the granular layer *in vivo *tend to occur in a range of 5 to 30 Hz [6,69]. This is within the range of slow frequencies, where the robustness effect was stronger. Additionally, the real synaptic weights for the PFs onto GoCs are not known. The simulation results predict that, if values of 10 to 30% are closer to physiological conditions than the 100% PF maximal synaptic weight used in the model, then the robustness effect would be stronger and not restricted to the first oscillatory cycles only. Additional experimental measurements will be necessary to confirm this prediction. Moreover, the cerebellar oscillations observed *in vivo *tend to be transient [5-7], which makes the effect of gap junctions on the initial oscillation cycles physiologically relevant.

Besides the robustness effect, we also observed a decrease in latency for the start of the oscillations after the activation of MF inputs. Because the frequency of oscillations did not change, the change in latency implies a change in the phase of oscillations. Because of the important function of the cerebellum in controlling the timing of movements and reflexes [70,71], such phase shifts can have important physiological consequences.

Recently two combined experimental modeling studies on the effect of GoC gap junctions on cerebellar oscillations presented contradictory results, which also differ from those in this study [28,29]. As mentioned above, a crucial difference in our experiment is that we investigated the behavior of the full circuit, including the PF feedback loop, whereas the other studies considered isolated GoC networks. In the accompanying experimental work, parasagittal slices were used where the PFs had been cut. An additional difference from the study of Dugué *et al*. [28] is our assumption that GoCs show spontaneous pacemaker firing, as observed by Forti *et al*. [40]. This discrepancy may result from differences in preparations. Forti *et al*. [40] recorded from cerebellar slices from 16 to 21-day-old Wistar rats whereas Dugué *et al*. [28] recorded from heterozygous 20 to 60 day-old GlyT2-eGFP C57/Bl6 mice, in which enhanced green fluorescent expression is controlled by the promoter of GlyT2, a glycine transporter. Besides the species and age differences, the transgenic manipulation might also have affected Lugaro neurons, which form a source of GoC inhibitory input [72]. Recently, Vervaeke *et al*. [29] suggested that gap junctions between GoCs could play a desynchronizing role when MF inputs are sparse. We confirmed that this is true for a network of isolated GoCs, but when the synaptic feedback loop was included, we recovered the robustness effect of gap junctions (Figure 12C), demonstrating that our results apply to a wide range of conditions.

Considering that inhibitory circuits with gap junctions showing oscillations are not exclusive to cerebellar neural networks [33,34,37,73], the robustness effect of gap junctions observed in our model may be a general mechanism present in other regions of the brain. In particular, our work suggests that results of experiments that block gap junctions should be interpreted with care; disappearance of oscillations does not necessarily imply that gap junctions are the essential underlying mechanism. Instead the oscillations may be primarily synaptically driven, but depend on gap junctions to smooth out disruptive noise caused by biological variability and other factors.

## Conclusions

Our modeling results suggest that gap junctions between GoCs do not cause the oscillations observed in the cerebellar granular layer under physiological conditions. Instead, they increase the robustness of these oscillations, which are driven by the synaptic feedback loop between GoCs and GCs. This effect is strongest for the first cycles of oscillation, and results in a permanent phase shift. The robustness effect of gap junctions may be generalized to other regions of the brain with synaptically driven oscillations.

## Competing interests

The authors declare that they have no competing interests.

## Authors' contributions

FMSS wrote the model scripts, ran the simulations, analyzed the data and wrote the manuscript. EDS has made substantial contributions to the concepts and design of the research, critically revised the manuscript, provided financial support, mentored the work and gave the final approval of the version to be published.

## Supplementary Material

Additional file 1 **Supplementary material**. This file contains all the supplementary figures and their respective legends. Click here for file
