# Supplementary material for: Robustness effect of gap junctions between Golgi cells on cerebellar cortex oscillations
Source: Neural Syst Circuits. 2011 Mar 1;1:7. doi: 10.1186/2042-1001-1-7 (PMC3278348; doi:10.1186/2042-1001-1-7)
Supplement: Additional file 1 — Supplementary material. This file contains all the supplementary figures and their respective legends. [file 2042-1001-1-7-S1.PDF]

Robustness Effect of Gap Junctions Between Golgi Cells on Cerebellar Cortex Oscillations

Fabio M Simoes de Souza[1], Erik De Schutter[1,2]

[1] Computational Neuroscience Unit, Okinawa Institute of Science and Technology, Okinawa 904-0411, Japan
[2] Theoretical Neurobiology, University of Antwerp, B-2610 Antwerpen, Belgium

Supplementary Material

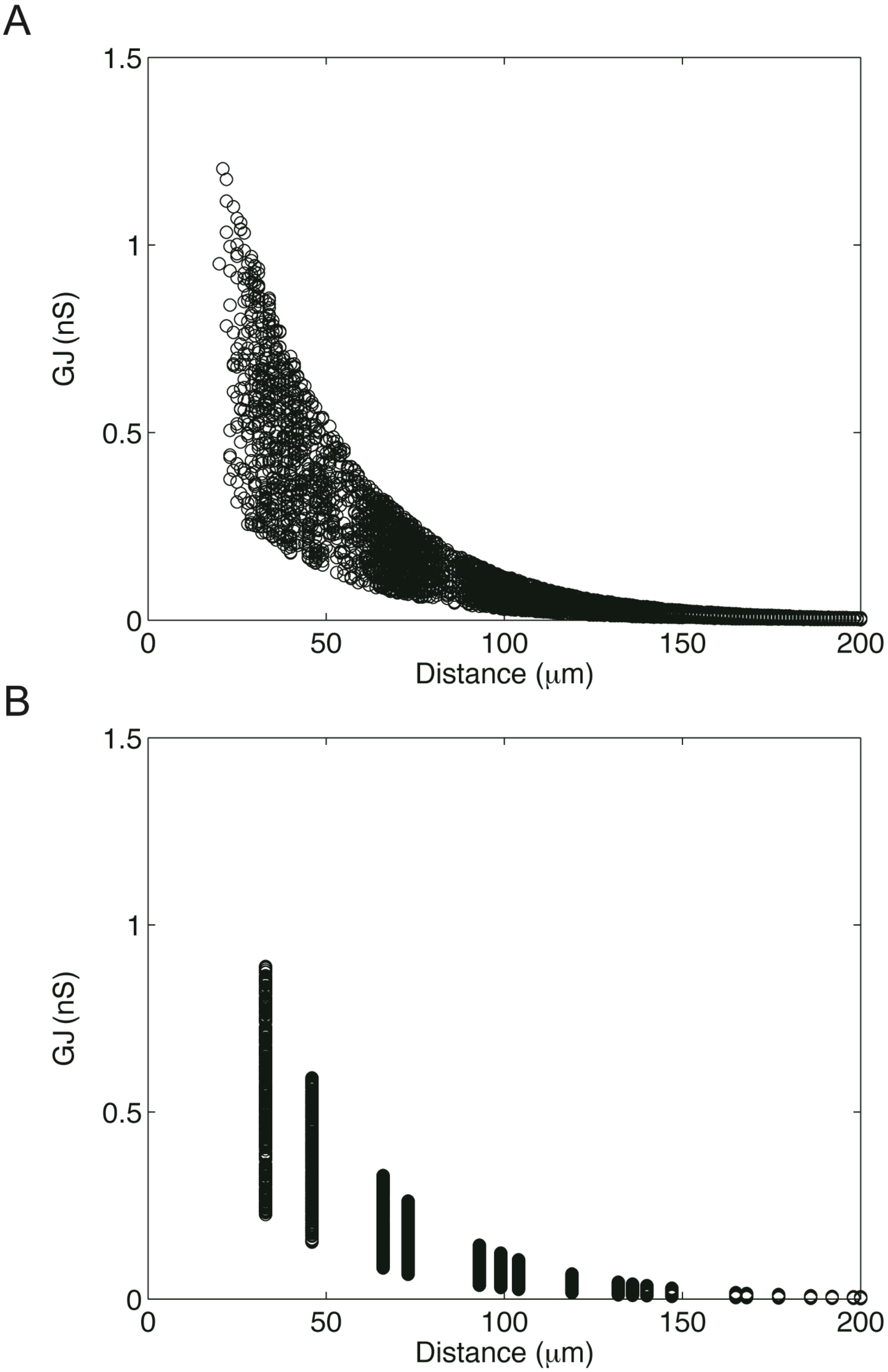

Figure S1 - Distribution of gap junction conductances (GJ (nS)) according to distance between GoCs.
A: Distribution of GJs in the presence of 20% spatial randomness in the original spatial coordinates (X and Y) of the GoCs. B: Distribution of GJs in the absence of spatial randomness in the X and Y coordinates of GoCs

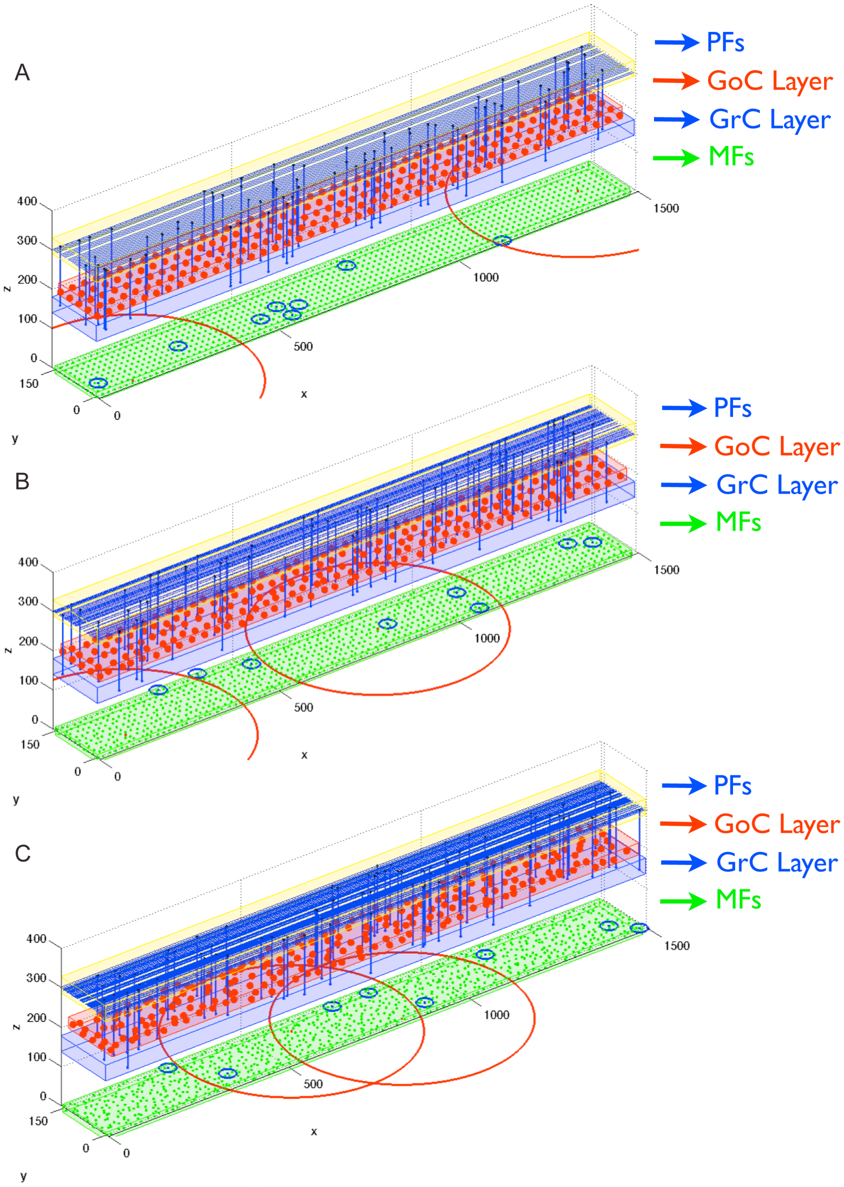

Figure S2 - Network topology with different levels of spatial randomness in the X and Y coordinates of the cells.
MFs are shown in green, GoCs in red, and GrCs and PFs in blue. Only 1% of the GrCs and PFs are displayed for better visualization. A: Network without spatial randomness. B: Network with 20 % randomness in the X and Y coordinates of each unit as used in most simulations. C: Network with 40 % of randomness in the X and Y coordinates of each unit. Note that the centers of the blue circles indicate the location of a few GrCs projected onto the MF layer, and the radius indicates the receptive field of these GrCs to MFs. The two red circles similarly indicate the receptive fields of two GoCs to MFs.

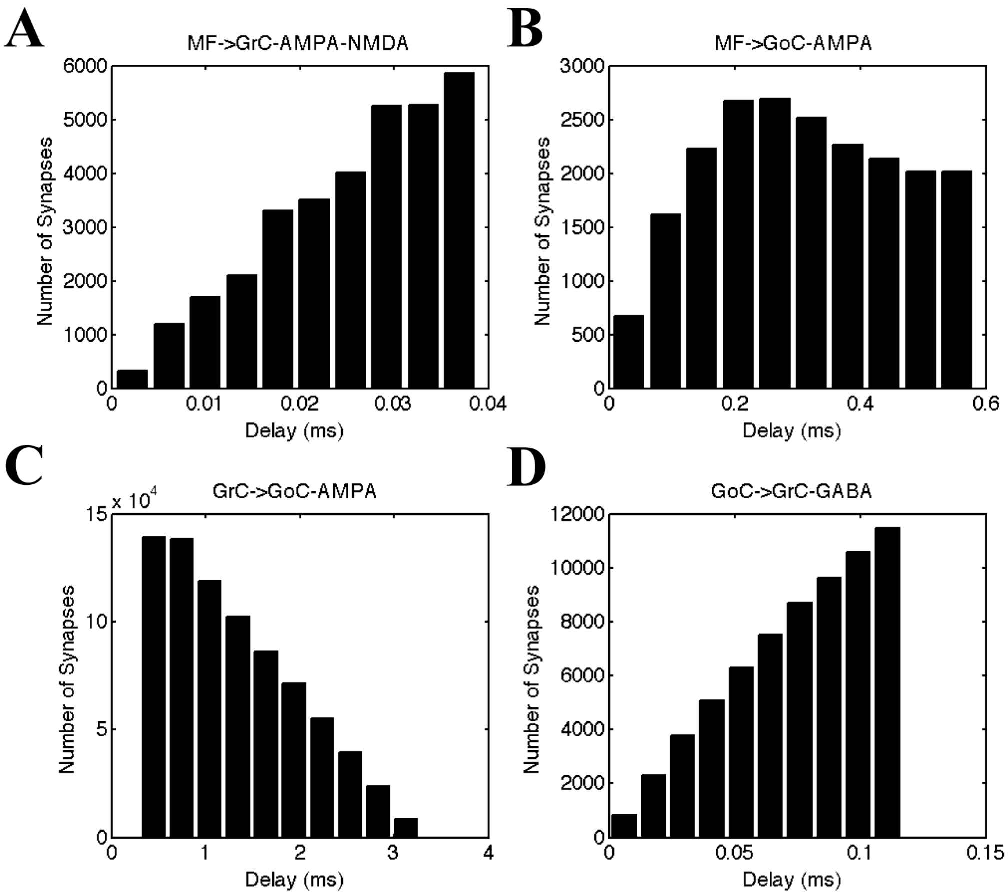

Figure S3 - Distribution of spike conduction times for each connection type.
A: MF to GrC AMPA and NMDA synapses. B: MF to GoC AMPA synapses. C: GrC to GoC AMPA synapses. D: GoC to GrC GABAergic synapses.

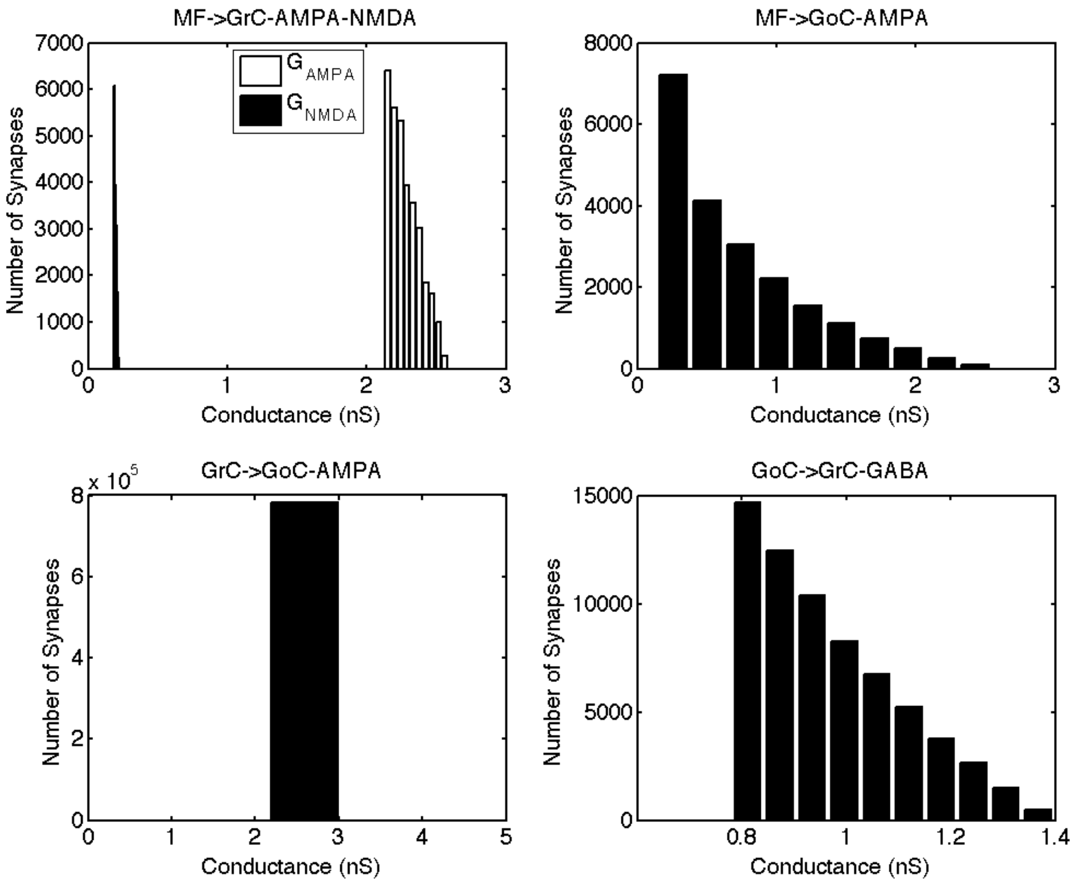

Figure S4 - Distribution of synaptic conductances for each connection type.
A: MF to GrC AMPA and NMDA synapses. B: MF to GoC AMPA synapses. C: GrC to GoC AMPA synapses. D: GoC to GrC GABAergic synapses.

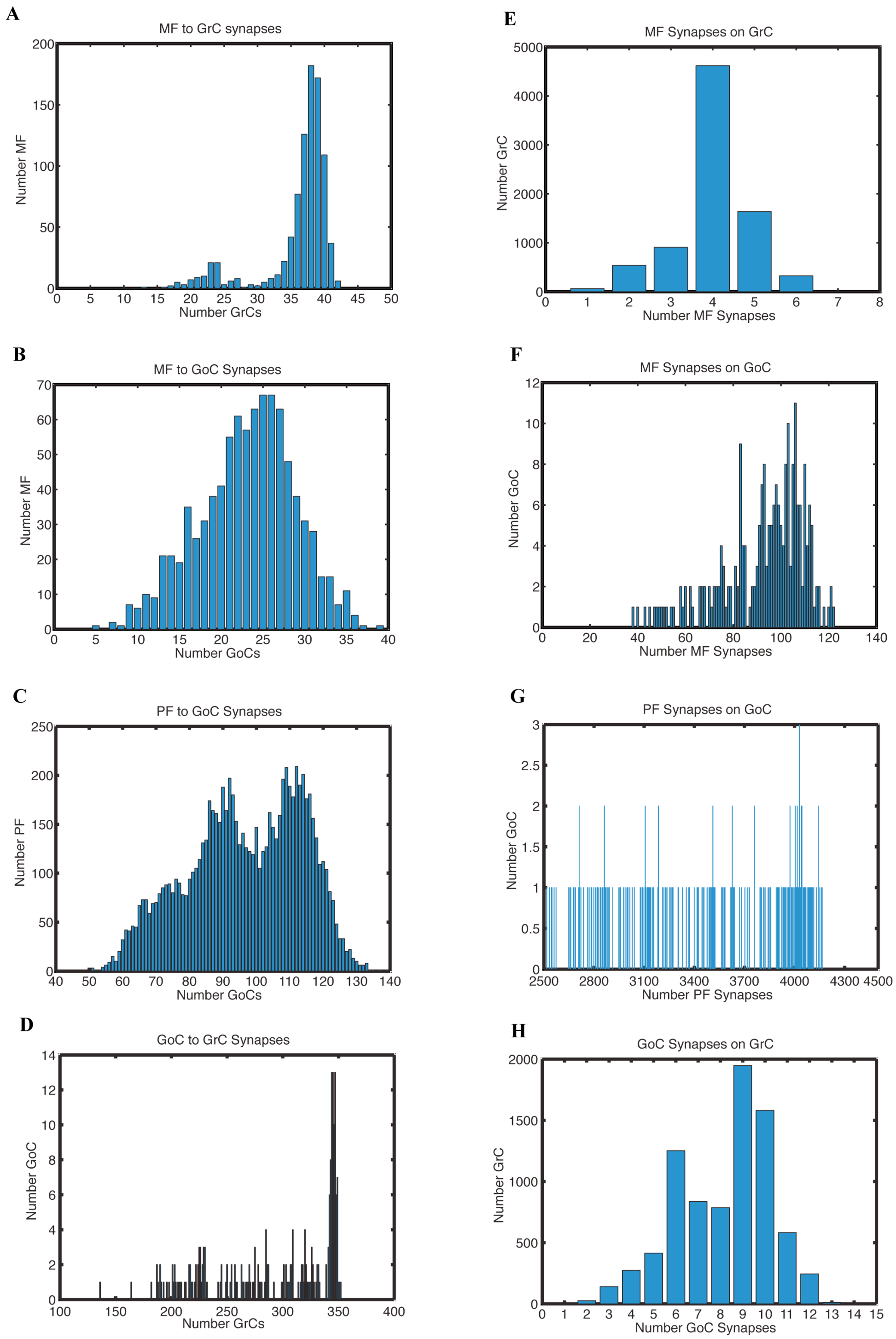

Figure S5 - Divergence and convergence patterns of connections in the neural network. A: divergence of MF to GrC synapses. B: divergence of MF to GoC synapses. C: divergence of PF to GoC synapses. D: divergence of GoC to GrC synapses. E: convergence of MF synapses on GrCs. F: convergence of MF synapses on GoCs. G: convergence of PF synapses on GoCs. H: convergence of GoC synapses on GrCs.

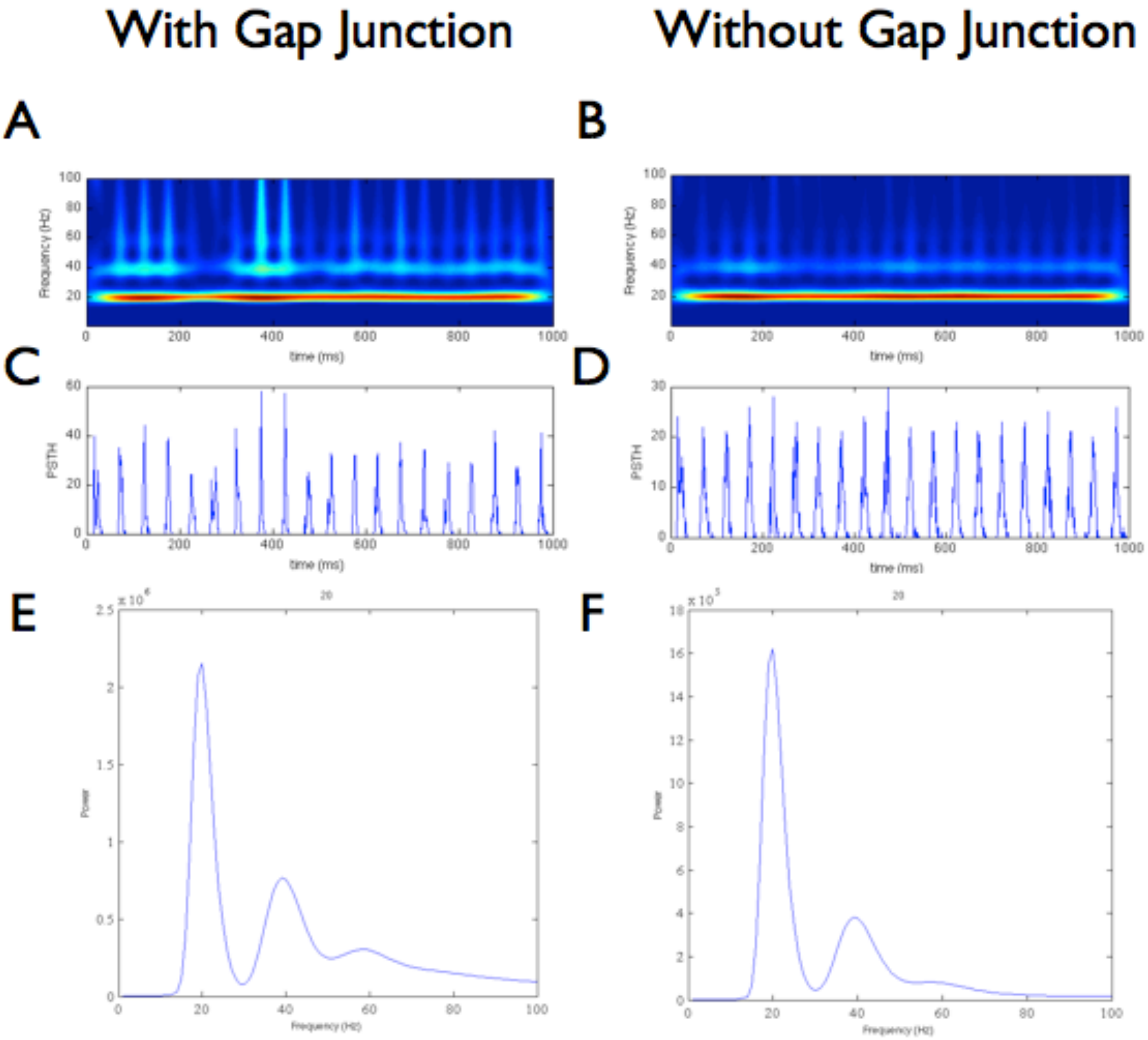

Figure S6 - Example of wavelet analysis of the oscillations.
This is the oscillatory response of the network to a sinusoidal stimulus at 20 Hz in the GoC layer. A and B: power spectrum evolving in time respectively for the PSTH of the GoC layer with and without gap junctions. Red colors represent higher powers and blue colors lower powers. There is a clear 20 Hz component along the whole simulation (0 to 1000 ms). C and D: respective PSTHs of the GoC layer with and without gap junctions. E and F: mean power spectrum across all the simulation time.

A

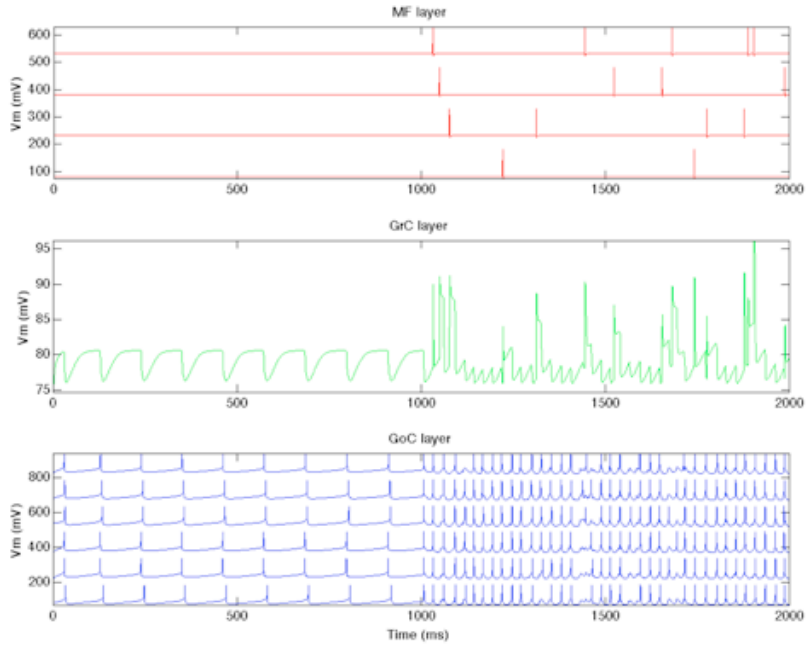

B

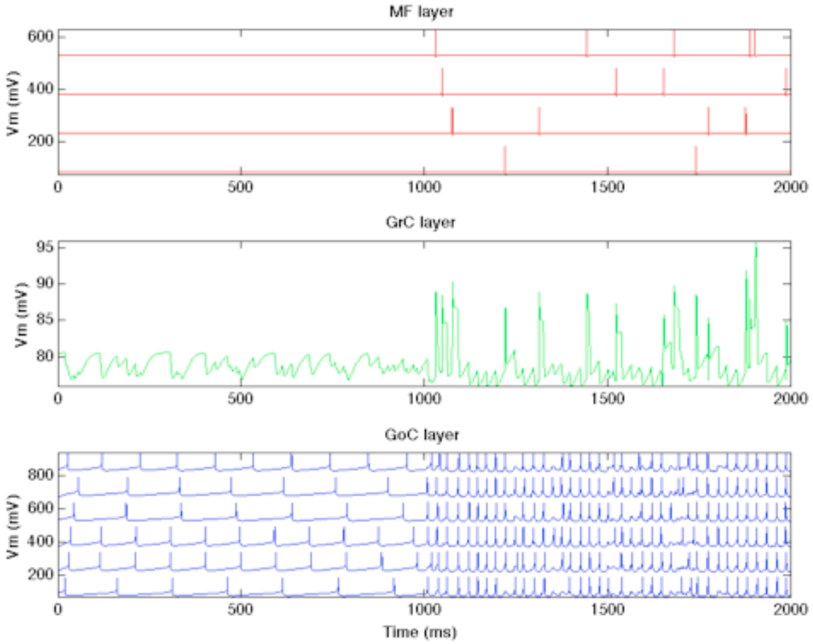

Figure S7 - Representative traces of firing patterns in the 3 different layers.
This simulation uses 100 % of the total synaptic weight for the PFs. A stimulation of the network with a MF MFR of 5 Hz is turned on at 1000 ms. The four MFs (red) show in the top panel are pre-synaptic to the GrC on the middle panel (green) and to the GoCs on bottom panel (blue). The presence of gap junctions does not affect the mean firing rate of the neurons. A: case with gap junctions between GoCs. B: case without gap junctions between GoCs.

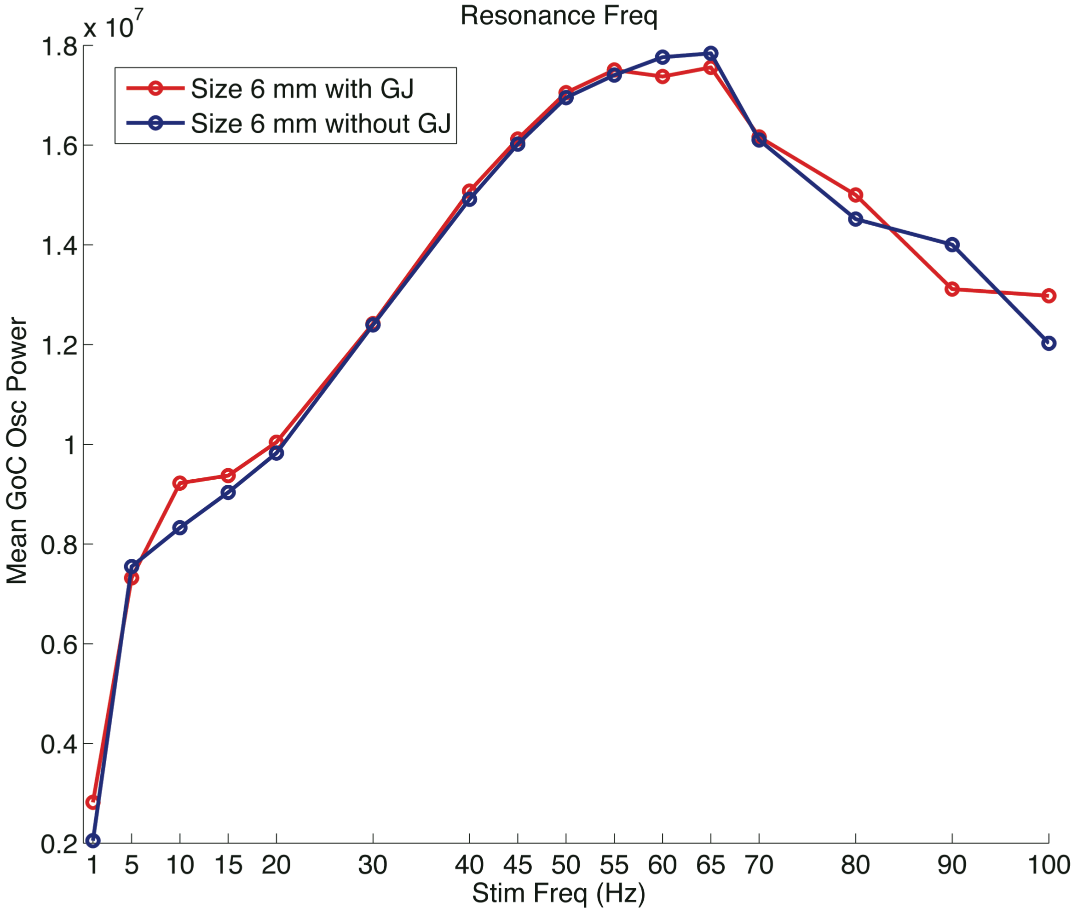

Figure S8 - Resonance frequency of the long network.
The network size was increased by four along the X axis (6 mm length), PFs have 100 % of the synaptic weight. The resonance frequency is around 65 Hz both in the presence (blue curve) and absence (red curve) of gap junctions between GoCs. There is a small increase in the power of the secondary peak at 10 Hz for the case with gap junctions.

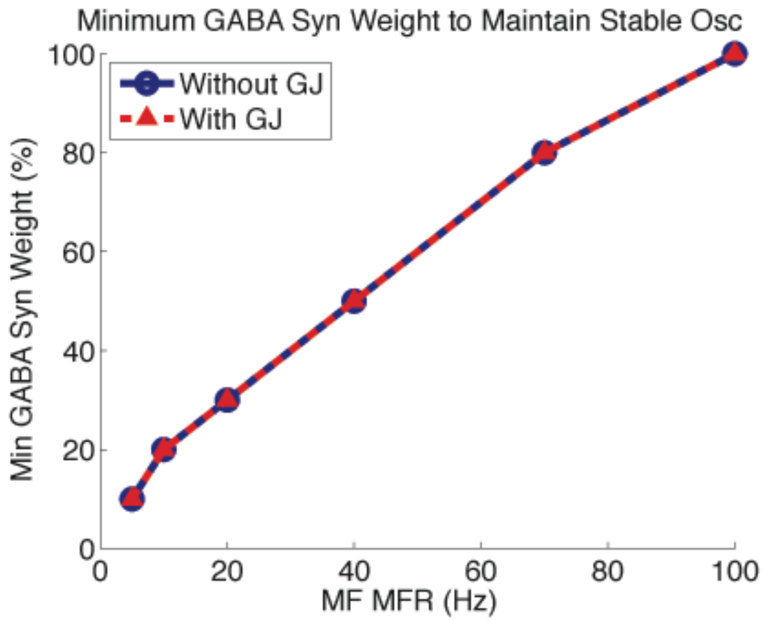

Figure S9 - Minimum GABAaR synaptic weight to maintain stable oscillations.
Stable oscillations are defined as continuing for the entire stimulation period (1000 to 2000 ms) in the network with 100 % of the PF synaptic weight. The GABAaR synaptic weight required to maintain stable oscillations increases almost lineally with the MF MFR, and it is independent of the presence or absence of gap junctions between GoCs.

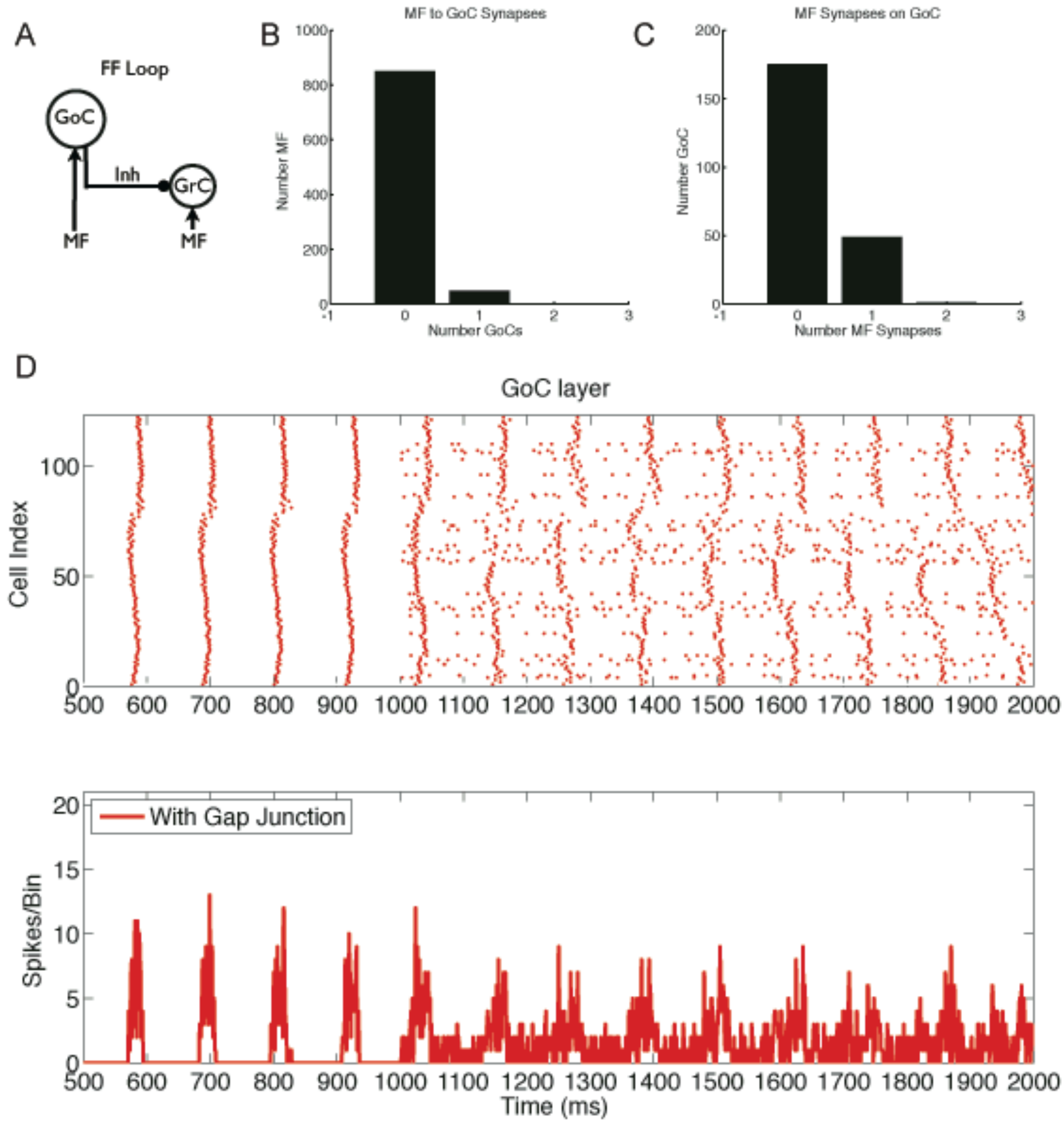

Figure S10 – Simulation of the network in the feedforward loop configuration and with sparse MF inputs at the MFR of 100 Hz.
A: Feedforward loop configuration – network without PF input to GoCs. B: Divergence pattern of MF to GoC connections with very sparse MF to GoC connectivity. Every MF connects to only 1 GoC, and most of the MFs are not connecting GoCs. The synaptic weight of the MF was increased 20 times to allow the synaptic excitation of GoCs by the sparse MFs. C: Convergence pattern of MF on GoC connections with very sparse MF to GoC connectivity. MFs excite 20 % of GoCs that receive 1 or 2 MF connections. It implies that 80 % of GoCs are not receiving any MF input. D: Raster plot (top panel) and PSTH (bottom panel) of the GoC layer with gap junctions and sparse MF inputs. Each dot in the raster plot is a spike. MF inputs at MFR of 100 Hz were turned on at the instant of 1000 ms. Note that gap junctions have a desynchronizing effect in the presence of the sparse MF inputs similar to the results of Vervaeke et al. (2010).

A

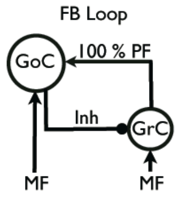

B

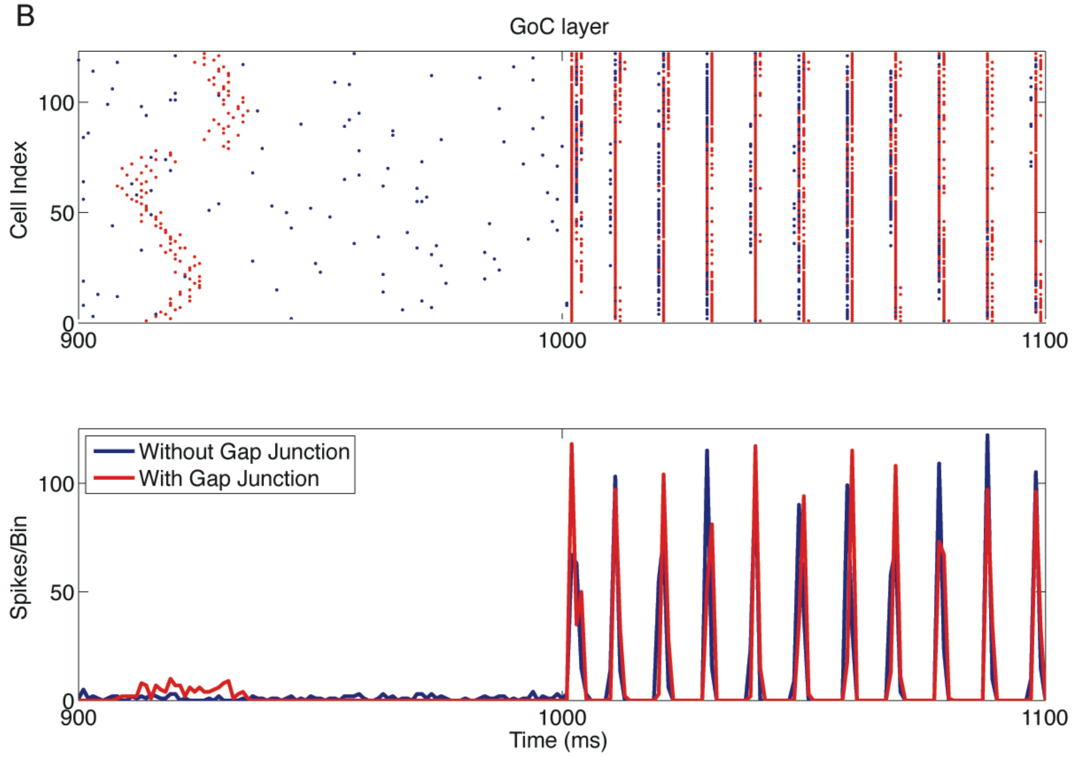

Figure S11 – Simulation of the network in the feedback loop configuration and with sparse MF inputs at the MFR of 100 Hz.
A: Feedback loop configuration with 100% of the synaptic weight of the PF inputs to GoCs. See Supplemental Figure 10B, C for sparse MF connectivity. B: Raster plot (top panel) and PSTH (bottom panel) of the GoC layer with gap junctions and sparse MF inputs. Each dot in the raster plot is a spike. MF inputs at MFR of 100 Hz were turned on at the instant of 1000 ms. Note that in the feedback loop configuration a network with sparse MF input still shows the normal robustness effect of gap junctions on the oscillations.

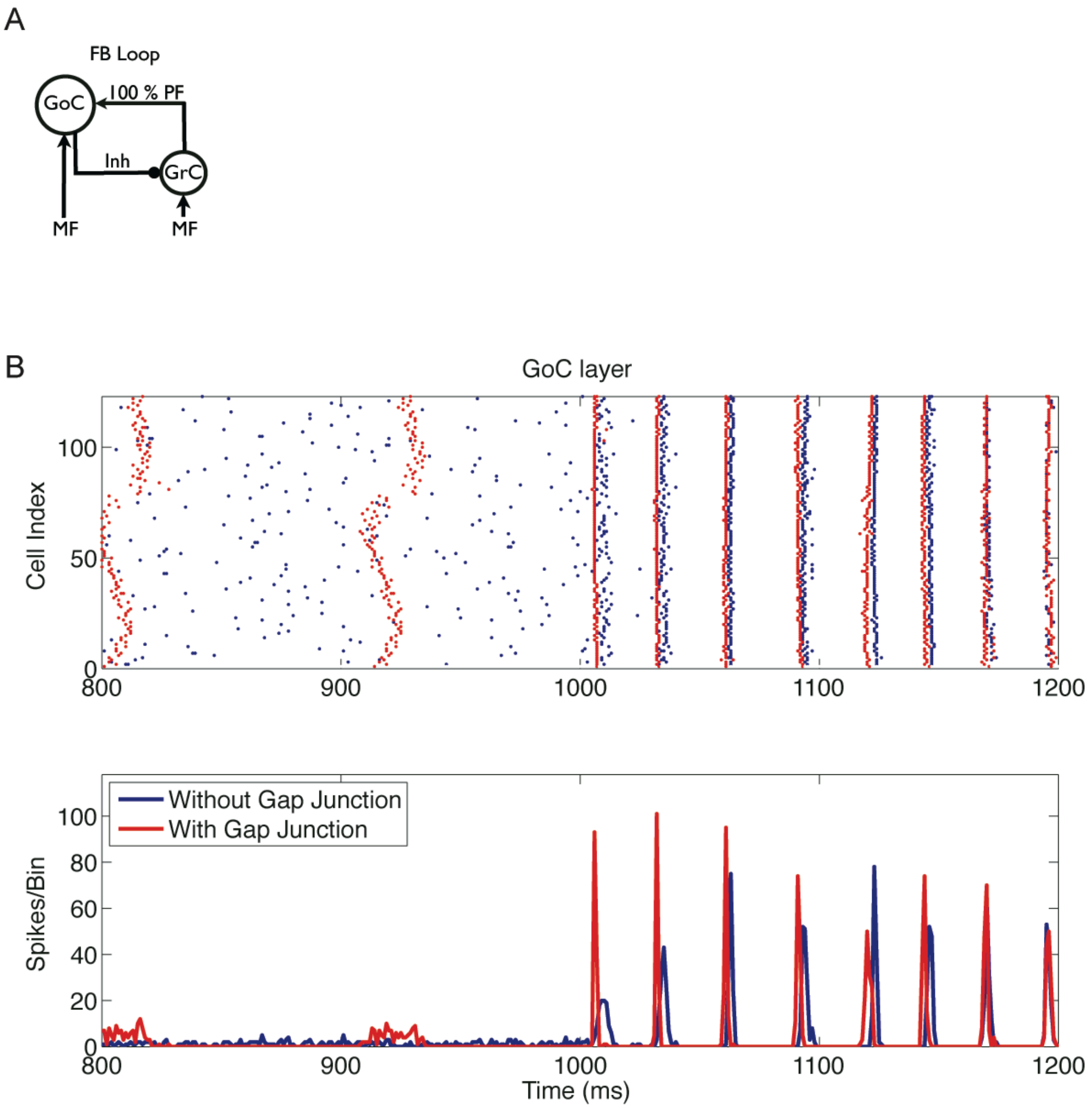

Figure S12 – Simulation of the network in the feedback loop configuration and with sparse MF inputs at the MFR of 5 Hz

A: Feedback loop configuration with 100% of the synaptic weight of the PF inputs to GoCs. See Supplemental Figure 10B, C for sparse MF connectivity. B: Raster plot (top panel) and PSTH (bottom panel) of the GoC layer with gap junctions and sparse MF inputs. Each dot in the raster plot is a spike. MF inputs at MFR of 5 Hz were turned on at the instant of 1000 ms. Note that in the feedback loop configuration a network with sparse MF input still shows the normal robustness effect of gap junctions on the oscillations.
